# Evaluation of Residual Stress in Metal Surface-Strengthened Layers Based on Specimen Deformation After Stripping Partial Substrate

**DOI:** 10.3390/ma19050860

**Published:** 2026-02-25

**Authors:** Dejun Li, Yuchi Lei, Jiangzhuo Ren, Xuewen Liu, Fengzhang Ren

**Affiliations:** 1 State Key Laboratory of Oil and Gas Equipment, CNPC Tubular Goods Research Institute, Xi’an 710077, China; lidejun352@163.com; 2 Key Laboratory of Road Construction Technology and Equipment of MOE, Chang’an University, Xi’an 710064, China; jxlyc@my.swjtu.edu.cn; 3 Ninth Oil Production Plant, Changqing Oilfield Branch Co., Yinchuan 750000, China; liuxw_cq@petrochina.com.cn; 4 School of Materials Science and Engineering, Henan University of Science and Technology, Luoyang 471003, China; renfz@haust.edu.cn

**Keywords:** residual stress, measuring, surface strengthening, stripping, substrate, metals

## Abstract

The present work aims to develop a deformation-based method for measuring the residual stress of the strengthened layer. The proposed method determines both the average residual stress in the strengthened layer and the layer’s thickness from the change in surface curvature after partial substrate removal. In order to examine the feasibility of the proposed method, the residual stress in the surface-strengthened layers and the layers’ thickness of 45 and 16Mn steel specimens subjected to shot-peening are measured using the proposed method and the widely recognized X-ray–electrochemical corrosion stripping method (X-/ECSM). The maximum differences in the residual stress and the thickness between the two methods for the two specimens are 19.7% and 17.6%, respectively. The results of two methods still show acceptable agreement, supporting the feasibility of the proposed method. The newly proposed method offers simpler specimen preparation, better machining accuracy control, and a more streamlined procedure compared to classical methods. However, it is inherently destructive, cannot determine residual stress distribution, and is unsuitable for specimens with complex geometries.

## 1. Introduction

Mechanical parts made of metal materials are often treated by surface-strengthening processes such as shot-peening, rolling, laser shock, induction heating, hardening, etc., to improve their performance during service, such as fatigue life and wear resistance [1,2]. This layer is caused by these surface-strengthening processes, and its material is the same as the substrate, unlike surface films and coating layers. There is usually residual stress in the surface-strengthened layer. The level of residual stress in the surface-strengthened layer is directly related to the performance of mechanical parts [3,4,5]. Thus, it is important to measure the magnitude of residual stress during the design and production of the parts.

There are many methods for measuring residual stress in the surface-strengthened layer, and they generally fall into two categories: non-destructive and destructive. The non-destructive methods have the obvious advantage of preserving the specimens. The main non-destructive methods include X-ray diffraction, neutron diffraction, X-ray synchrotron radiation, and ultrasonic and magnetic methods. X-ray diffraction is often used in production sites and laboratories because of its convenient operation and its high accuracy; however, the measurement depth is only approximately 10–30 μm [6,7]. Neutron diffraction and X-ray synchrotron radiation methods can measure residual stresses at great depths [8,9], but their efficiency is low and their measuring equipment is more expensive and very scarce. The ultrasonic and magnetic methods can detect the residual stress both inside and at the surface layer of the sample [10,11], and their measuring equipment is easy to carry and can be conveniently used in production sites. However, they also have obvious shortcomings; for example, a calibration experiment is needed to measure residual stress by the ultrasonic method, and the magnetic method can only be used for magnetic materials. Moreover, their results are easily affected by the material’s structure, the surface roughness of the specimen and the ambient temperature [12,13].

The main destructive methods include X-ray diffraction combined with electrochemical removal of a layer, drilling holes, crack compliance, the contour method and the stripping method [14]. All these methods can be used to measure the internal distribution of stress in a metal sample, and the cost of these tests is low. The X-ray diffraction method combined with a process of stripping by electrochemical corrosion (X-ray–electrochemical corrosion stripping method, X-/ECSM) is more widely used to measure the residual stress in the modified surface layer [15,16]. However, the depth of corrosion is not easy to control and measure in actual operations. Moreover, the removal of a layer by electrochemical corrosion will result in the redistribution of internal stress in the sample, so there is a slight difference between the residual stress measured by X-/ECSM and the initial residual stress in the sample. In order to compensate for the changes in the residual stresses caused by layer removal, the FEM (finite element method) correction method was used to correct the stress values obtained by X-/ECSM [17], but the correction method is complex. The hole-drilling methods can be further divided into conventional hole-drilling, the ring-core method and the deep hole method [18,19]. The accuracy of the hole-drilling method will be affected by the drill’s eccentricity and additional stress from machining the hole [20]. The accuracy of the crack compliance method is generally higher than that of the hole-drilling method and the stripping method, but its accuracy is affected by the size effect [21,22]. The contour method is a method of measuring residual stress based on sectioning relaxation, which can be used to obtain a two-dimensional distribution of stress relative to the cutting plane. By comparison, other methods, such as hole-drilling and stripping, give a one-dimensional distribution of stress. The main difficulty of the contour method is the requirements for high-precision cutting of the section, especially for smaller sections [23]. The classical stripping method is mostly used to obtain the internal residual stress of surface layer of flat plate specimens or those with a definite curvature radius. In this method, the residual stress layer is artificially divided into several layers and then removed layer by layer. Assuming that the residual stress is uniformly distributed in each layer of a specimen and each layer has its own residual stress, when a layer is stripped off, the constraint of that layer on the other layers is eliminated and internal stress is redistributed due to the self-equilibrating nature of internal stress, which causes deformation of the rest of the specimen. According to the measured deformation of the rest of the specimen, the residual stress distribution parallel to the surface can be approximately deduced by finite element simulation [24].

In our work, a new and simple measurement method of residual stress is designed, named the reducing substrate constraint method (RSCM). It resembles the classical stripping method but differs in the removing part. Unlike the classical stripping method which removes part of the surface-strengthened layer, it removes part of the substrate (surface residual stress still remains, but it will be redistributed). In the classical stripping method for removing a portion of the surface-strengthened layer (since the strengthened layer is typically quite thin, the stripped layer is thinner), precise thickness control is challenging due to the need for small removal depth. In contrast, with the RSCM, it is easier to achieve accurate control when removing thicker substrate sections. The RSCM determines the average residual stress and its effective depth (corresponding to the strengthened layer thickness) in the original strengthened layer by analyzing the stress redistribution and resulting deformation of the rest of the specimen after partial substrate removal. It has the advantages of convenient operation and intuitive measurement process. In order to examine the feasibility of the new method, the residual stress in the surface-strengthened layers of 45 (Chinese grade, equivalent to ASTM/AISI 1040; Standard Guide for Specifying Harmonized Standard Grade Compositions for Wrought Carbon, Low-Alloy, and Alloy Steels; Publisher: ASTM International, West Conshohocken, PA, USA, 2022) and 16Mn (Chinese grade, equivalent to ASTM/AISI SA-516GR65; Standard Specification for Pressure Vessel Plates, Carbon Steel, for Moderate- and Lower-Temperature Service; Publisher: ASTM International, West Conshohocken, PA, USA, 2023) steels treated by shot-peening are measured using the new method and X-/ECSM, and the results of the two methods are compared. Although measurement of the residual stress in the surface-strengthened layer using X-/ECSM is very time-consuming and inefficient, the results of this method are widely recognized. The measured result of X-/ECSM is used as the standard of evaluation to test the feasibility of the new method.

## 2. Process and Principles of the New Method (RSCM) for Measuring Residual Stress

The principle behind the RSCM is simply as follows: when a large proportion of the substrate of the surface-strengthened specimen is stripped off, the remaining part of the specimen will visibly bend and deform. The surface’s radii of curvature of the surface-strengthened layer are measured both before and after partial removal of the substrate. The measured radius of curvature and the thickness of the remaining part of the specimen are put into the formula derived using linear elasticity, and then the average residual stress and its effective depth (i.e., the thickness of the surface-strengthened layer) can be calculated.

### 2.1. Cutting the Specimens and Measurement of the Deformation

To calculate the average residual stress and its effective depth, measurement data from two specimens surface-strengthened by the same process will be required. The two specimens must retain different substrate thicknesses after most of the substrate has been stripped. Based on these requirements, two specimens are prepared and measured as follows.

**Figure 1:**
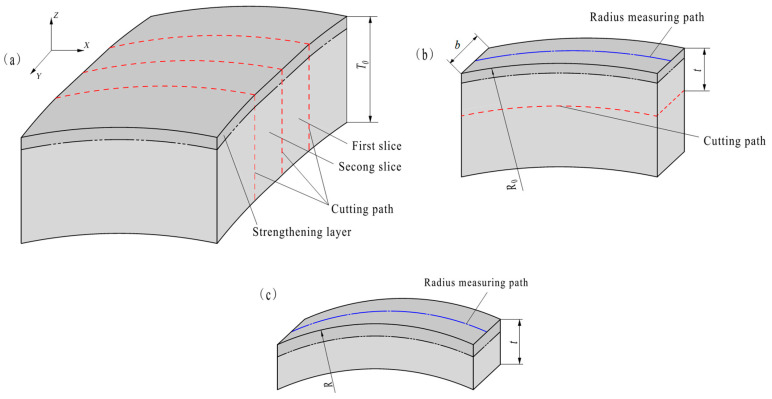
(**a**) Path of cutting the narrow slices; (**b**) cut-out narrow slice; (**c**) cut-out strip.

Two narrow slices with a small width *b* are cut from a surface-strengthened metal plate with a thickness *T*_0_ by wire electrical discharge machining, as shown in Figure 1a. Due to multidirectional residual stresses, the slice surface exhibits non-flatness in its *Y* direction profile. Nevertheless, its small *Y*-width allows treating it as flat. Because the width of the narrow slices (in the *Y* direction) is relatively small (see Figure 1b), the radius of the curvature (*R*_0_) of the surface of the strengthened layer is considered to be the same everywhere along the *Y* direction (width). *R*_0_ can be measured along the middle line in the direction of the width of the surface (see Figure 1b).

A strip containing the strengthened layer is cut from a narrow slice by wire electrical discharge machining. (It is generally believed that wire electrical discharge machining does not induce residual stresses.) The thickness (*t*) of the strip has to be much larger than the effective depth (*t*_0_) of the residual stress (*t* >> *t*_0_, after estimating *t*_0_), or else the internal stress cannot be assumed to be the same throughout the depth. The *t*/*t*_0_ ratio should be as large as possible, but not too large. The larger the ratio, the less noticeable the deformation becomes, making it increasingly difficult to measure the deformation accurately. When the strip is cut from a narrow slice on a wire electrical discharge machine, the clamping position of the narrow slice cannot be on the strip to avoid interference with the free deformation of the strip. That is, the clamping position is on the stripped part of the substrate (i.e., the clamping position is on the part below the cutting path in Figure 1b). The radius of curvature of the cutting path is *R*_0_-*t*. The cut-out strip is as shown in Figure 1c. Because most of the substrate of the slice is cut off, the radius of the curvature of the surface of the strengthened layer changes. The radius of curvature (*R*) of the surface of the strengthened layer is also considered to be the same everywhere along the *Y* direction, and is measured along the middle line in the direction of the width of the strip’s surface. During cutting, the thickness (*t*) of the two strips should be significantly different and is far less than *T*_0_ (i.e., *t* << *T*_0_). When *t* << *T*_0_, the curvature radius of the cutting path remains essentially constant during strip cutting, resulting in consistent thickness along the entire strip length. The values of *R* and *t* of the two strips are plugged into formulas based on the principles of elastic mechanics, and then the average residual stress *σ*_0_ and its effective depth *t*_0_ in the strengthened layer are calculated.

### 2.2. Formulas for Stress

The residual stress in a film on a substrate can be calculated using the Stoney formula [25]. However, this formula is applicable to layered composites with a thin substrate and an extremely thin film. In contrast, surface-strengthened mechanical parts and specimens are typically thick, and the deformation caused by surface strengthening is minimal, making the Stoney formula unsuitable for calculating residual stress. In our work, deformation is amplified by reducing the substrate thickness. Nonetheless, the Stoney formula remains inappropriate even for specimens with a thinner retained substrate, as evident from its derivation process. Therefore, a new formula for calculating residual stress needs to be derived.

**Figure 2:**
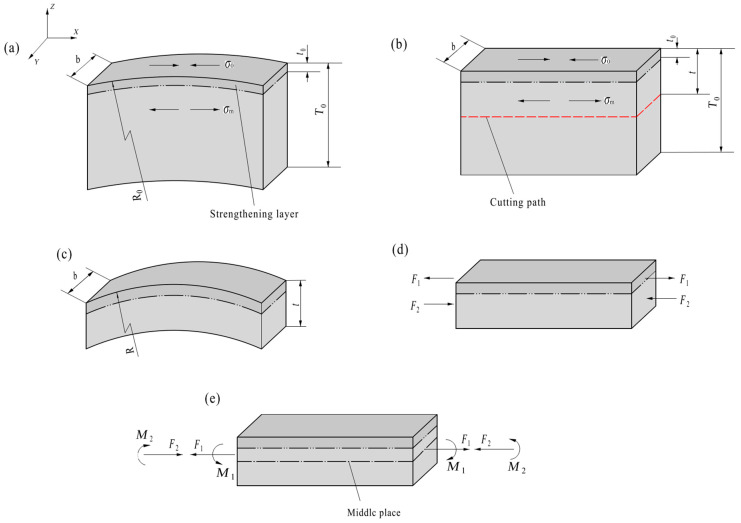
(**a**) Cut-out narrow slice and its internal stresses, (**b**) the presumed straight narrow slice, (**c**) bent strip cut from straight narrow slice, (**d**) the presumed straight strip, and (**e**) force translation of the straight strip.

As shown in Figure 2a, *T*_0_, *b* and *R*_0_ denote the thickness, the width and radius of curvature at the surface of the narrow slice cut from the surface-strengthened metal plate, respectively. The average residual stress in the surface-strengthened layer, its effective depth (i.e., the thickness of the surface-strengthened layer) and the average internal stress in the substrate (the part below the surface-strengthened layer) are represented by *σ*_0_, *t*_0_ and *σ*_m_, respectively.

According to the equilibrium conditions of forces acting on the narrow slice, the relationship between *σ*_m_ and *σ*_0_ can be given as follows:

(1) $$\sigma_{m}=\frac{t_{0}}{T_{0}-t_{0}}\sigma_{0}$$

For the sake of analysis, the narrow slice is assumed to be straight and has the same internal stress as the bent slice, as shown in Figure 2b. The strip cut from the straight narrow slice will appear to be bent, as shown in Figure 2c. When a significant portion of the substrate (the area below the cutting path in Figure 2b) is removed, it is assumed that the internal stress of the remaining strip (the area above the cutting path in Figure 2b) remains unchanged. This results in an imbalance of internal stress within the strip, causing it to bend and subsequently re-balance its internal stress. The radius of curvature at the surface of the strengthened layer of the bent strip is represented by the symbol *R*.

A straight strip, identical in thickness and width to the bent strip and free of internal stress, is artificially compartmentalized into two layers: an upper layer with the same thickness as the surface-strengthened layer, and a lower layer. To achieve the same degree of bending as the bent strip, forces *F*_1_ (=*σ*_0_*t*_0_*b*) and *F*_2_ (=*σ*_m_(*t* − *t*_0_)*b*) are applied at the midpoints of the end faces of the upper and lower layers of the straight strip, respectively (the bent strip is shown in Figure 2c). Figure 2d shows a straight strip subjected to the forces *F*_1_ and *F*_2_, as described above. According to the force translation theorem, when *F*_1_ and *F*_2_ are translated to the middle positions of the end face of the strip, two extra bending moments *M*_1_ and *M*_2_ must be attached (see Figure 2e). *M*_1_ and *M*_2_ must satisfy the following:

(2) $$M_{1}=F_{1}(\frac{t}{2}-\frac{t_{0}}{2})=\sigma_{0}t_{0}b\frac{t-t_{0}}{2}$$

(3) $$M_{2}=F_{2}\frac{t_{0}}{2}=\sigma_{m}(t-t_{0})b\frac{t_{0}}{2}=\sigma_{0}b\frac{{t_{0}}^{2}(t-t_{0})}{2(T_{0}-t_{0})}$$

**Figure 3:**
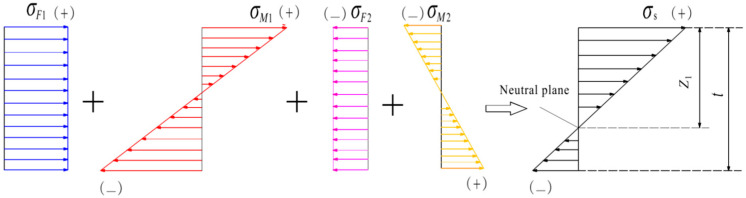
The stress distribution on the cross-section of the straight strip (stress direction (arrow) and type (color)).

The internal stresses on the cross-section of the straight strip caused by *F*_1_, *M*_1_, *F*_2_ and *M*_2_ are represented by *σ_F_*_1_, *σ_M_*_1_, *σ_F_*_2_ and *σ_M_*_2_, respectively. The distributions of these internal stresses and their synthetic stress (*σ*_s_) are shown in Figure 3. The synthetic stresses at the top edge (*σ*_ts_) and the lower edge (*σ*_ls_) of the cross-section are calculated as follows:

(4) $$\sigma_{\mathrm{ts}}=\sigma_{F1}+\sigma_{M1(\max)}-\sigma_{F2}-\sigma_{M2(\max)}=\frac{\sigma_{0}t_{0}(4T_{0}t-3T_{0}t_{0}-6t_{0}t+6{t_{0}}^{2}-t^{2})}{t^{2}(T_{0}-t_{0})}$$

(5) $$\sigma_{\mathrm{ls}}=\sigma_{F1}-\sigma_{M1(\max)}-\sigma_{F2}+\sigma_{M2(\max)}=\frac{\sigma_{0}t_{0}}{t^{2}}(3t_{0}-2t+\frac{4t_{0}t-t^{2}-3{t_{0}}^{2}}{T_{0}-t_{0}})$$

where *σ_F_*_1_ = *F*_1_/(*bt*), *σ_F_*_2_ = *F*_2_/(*bt*); *σ_M_*_1(max)_ and *σ_M_*_2(max)_, respectively, are the maximum of *σ_M_*_1_ and *σ_M_*_2_; *σ_M_*_1(max)_ = *M*_1_/*W*, *σ_M_*_2(max)_ = *M*_2_/*W*, *W* is the section modulus during bending and *W* = *bt*^2^/6.

According to Figure 3 and Equations (4) and (5), the distance (*z*_1_) between the neutral plane and the top edge is calculated as follows:

(6) $$z_{1}=\frac{t(4tT_{0}-3t_{0}T_{0}-6t_{0}t+6{t_{0}}^{2}-t^{2})}{6(t-t_{0})(T_{0}-2t_{0})}$$

Because the straight strip acted upon by the forces *F*_1_ and *F*_2_ will achieve the same degree of bending as the bent strip cut from the straight narrow slice, the radius of the curvature of the upper surface of the bent strip is also *R*. *R* should be much larger than *z*_1_. The relationship of *σ*_ts_, *R* and *z*_1_ is as follows:

(7) $$\sigma_{\mathrm{ts}}=E\varepsilon_{\mathrm{ts}}=E\frac{z_{1}}{R-z_{1}}\approx E\frac{z_{1}}{R}$$

where *E* is the elastic modulus and *ε*_ts_ is the strain of the upper surface of the bent strip.

Substituting *σ*_ts_ (Equation (4)) and *z*_1_ (Equation (6)) into Equation (7), the formula for computing *σ*_0_ is derived as follows:

(8) $$\sigma_{0}=E\frac{t^{3}(T_{0}-t_{0})}{6t_{0}(t-t_{0})(T_{0}-2t_{0})}\frac{1}{R}$$

In the analysis above, the narrow slice is assumed to remain unbent. However, it is bent out of the plane of the original plate; it has a radius of curvature *R*_0_ as described above. When accounting for the original deformation *R*_0_, and given that both our formulation and the Strony formula (or the revised Strony formula) derivation fundamentally rely on force equilibrium principle with essentially identical procedural logic, *σ*_0_ can be approximated by applying the revising way of Berry and Pritchet (1990) [25] for the Strony formula as follows:

(9) $$\sigma_{0}=E\frac{t^{3}(T_{0}-t_{0})}{6t_{0}(t-t_{0})(T_{0}-2t_{0})}(\frac{1}{R}-\frac{1}{R_{0}})$$

In their revision way for the Strony formula, if the substrate has an initial curvature radius *R*_0_ before film deposition, the term 1/*R* in the Stroney formula is replaced by 1/*R* − 1/*R*_0_, where *R* is the curvature radius of the composite beam (substrate with deposited film) after deposition.

### 2.3. Identification of the Residual Stress and the Thickness of the Surface-Strengthened Layer

By substituting the measured *t* and *R* of both bent strips cut from two narrow slices into Equation (9), two equations are obtained. An equation group composed of the two equations is solved to obtain *σ*_0_ and *t*_0_.

The surface-strengthening process usually creates a layer of compressive stress, which inevitably results in the bending of the strip towards the side of the substrate; therefore, it must be true that *σ*_ts_ > 0, *σ*_ls_ < 0 and *z*_1_ > 0 in Equations (4)–(6). Moreover, *t* must be much larger than *t*_0_. The calculated values of *σ*_0_ and *t*_0_ must satisfy this condition; if not, the path of cutting needs to be adjusted.

## 3. Testing the Feasibility of the New Method (RSCM)

X-/ECSM is used to test the feasibility of the new method. That is, for the same surface-strengthened specimen treated by shot-peening, the residual stress in the surface-strengthened layer is measured by the X-/ECSM and the new method (RSCM). The result of X-/ECSM is used as the standard of evaluation to test the feasibility of the new method.

### 3.1. Preparation of Shot-Peened Specimens of Two Steels

Two plate specimens are cut from a quenched and tempered plate of 45 steel (Chinese grade) and a normalized plate of 16Mn steel (Chinese grade). Their average surface roughness is about 0.10 μm and their dimension is 120 mm × 120 mm × 18.5 mm after grinding. A surface with a size of 120 mm × 120 mm is shot-peened. The shot-peening device is a turntable shot-blasting cleaning machine (Q765, Yancheng Linater Machinery Manufacturing Co., Ltd., Yancheng, China). The shots and the velocity and time of shot-peening are cast steel shots 1.5–2.0 mm in diameter, 18.20 m·s^−1^ and 30 min, respectively. During shot-peening of the specimen, not only the upper surface but the four lateral sides are also exposed to the shot stream. After shot-peening, the 1.5 mm thick edges of the specimens are cut off (i.e., the edges containing the hardened layer are cut off; it can be inferred that the hardened layer is definitely less than 1.5 mm) and their final size is 117 mm× 117 mm× 18.5 mm. The shot-peening device features a large rotating specimen platform and a prolonged peening duration, resulting in a uniform hardened layer and consistently distributed stress across the upper surface of the specimen.

### 3.2. Measuring the Residual Stress via X-/ECSM

The devices used for X-/ECSM are a portable X-ray stress instrument (XSTRESS3000, Stresstech Oy, Vaajakoski, Finland) and a portable electrolytic polishing and etching apparatus (Struers Movipol-3, Struers, Champigny sur Marne, France). The test point is located on the surface of the surface-strengthened layer of the shot-peened specimen. The material is stripped by electropolishing to create a circular flat surface with a diameter of 10 mm. The voltage and current of stripping and polishing are 10 V and 1.5 A, respectively. The electrolyte solution is saturated salt water. The depth of corrosion stripping at each increment in the electropolishing test is controlled by the electrolytic polishing time and adjusted with a depth micrometer (0.01 mm precision).

**Figure 4:**
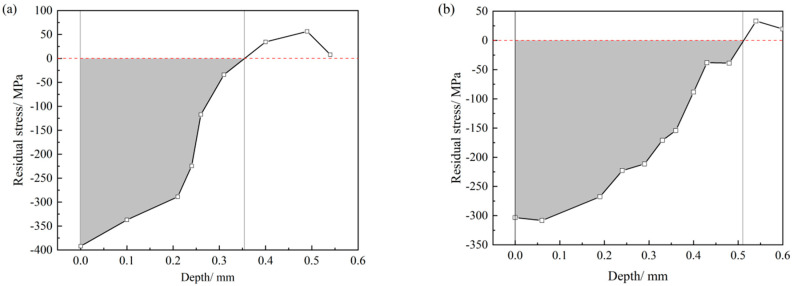
Variation in the average residual stress of both directions with the depth. Shot-peened specimens of (**a**) 45 and (**b**) 16Mn steel.

The residual stresses (parallel to the surface) of the two mutually vertical directions (0° and 90°) in the exposed surface after each increment in the corrosion depth are measured by the side inclining method with CrKα radiation. The measured distributions of residual stress along the depth are shown in Table 1 (the shot-peened specimen of 45 steel) and Table 2 (shot-peened specimen of 16Mn steel). Since the material removal depth for each electrochemical removal is relatively small, the difference between the residual stress measured by X-/ECSM and the original residual stress at the same depth is negligible. Therefore, no modification of the measured residual stress is performed. In line with the data in Table 1 and Table 2, the variation in the average residual stress in the two directions with the depth is plotted in Figure 4. The acting thickness (*t*_0_) of the residual stress is the depth at which the residual stress goes to zero in Figure 4. The average residual stress *σ*_0_ is calculated using the following equation:

(10) $$\sigma_{0}=\frac{\int_{0}^{t_{0}}\sigma_{d}dt}{t_{0}}$$

where *σ*_d_ is the residual stress distributed along the depth.

As shown in Figure 4, the *t*_0_ of the shot-peened specimens of 45 steel and 16Mn steel are 0.35 mm and 0.51 mm, respectively. In light of the data in Table 1 and Table 2 and Figure 4, and using Equation (10), the *σ*_0_ of the shot-peened specimens of 45 steel and 16Mn steel are calculated to be −256 and −198 MPa, respectively.

### 3.3. The Residual Stress Measured by the New Method (RSCM)

The shot-peened specimens are cut and measured according to the procedures described in Section 2. The cuts are made on a wire electrical discharge machine (DK7732, Changzhou Beichen CNC equipment Co., LTD, Changzhou, China), with the feeding rate and diameter of the wire being 6 m·s^−1^ and 0.18 mm, respectively. The thickness is measured with a vernier caliper with a precision of 0.02 mm. The radius of the curvature is measured with a Bearing Measurement System PGI 820 stylus profilometer with a precision of 0.1 μm (Taylor Hobson, Leicester, UK). After positioning and securing the specimen on the profilometer’s stage, the measurement stylus automatically traverses along the midline in the width direction of the specimen. The stylus tip has a radius of 0.02 mm.

**Figure 5:**
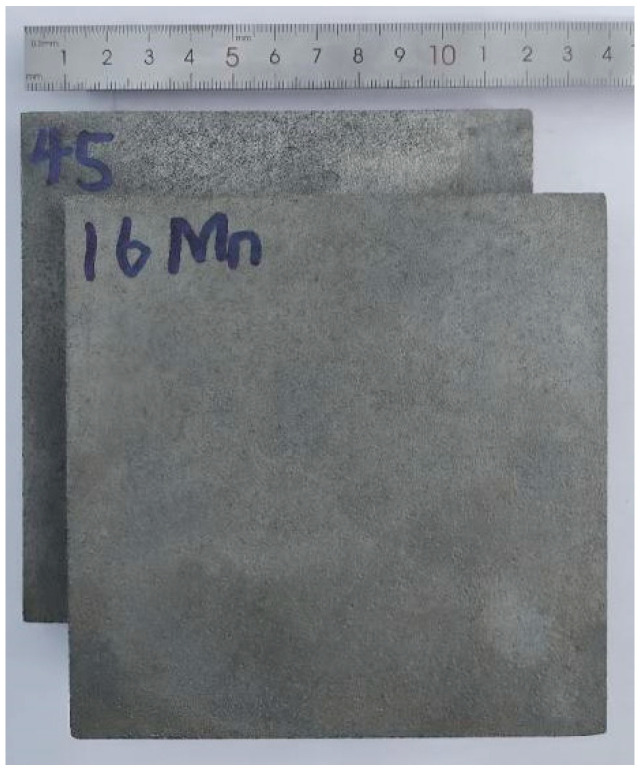
Shot-peened specimens.

**Figure 6:**
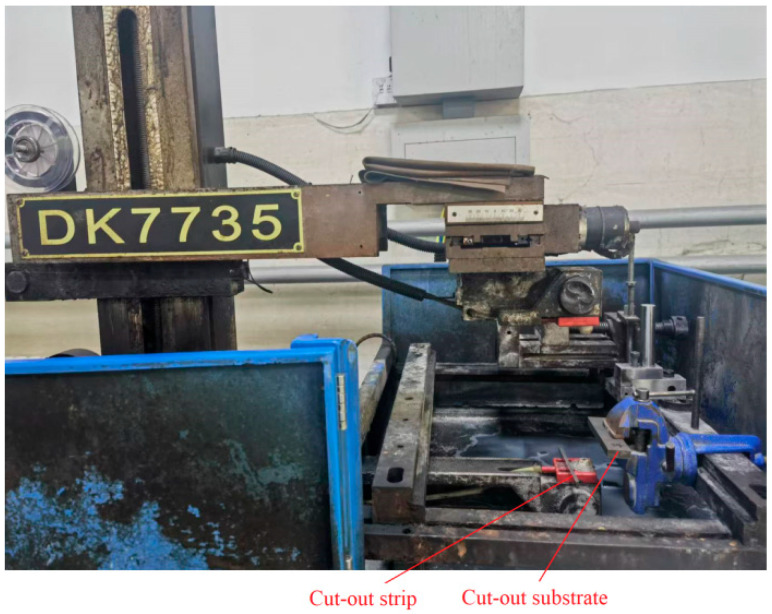
Cutting of slice.

**Figure 7:**
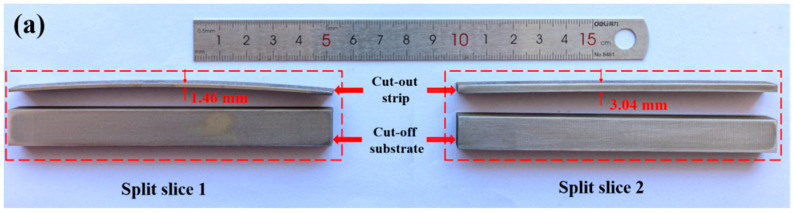

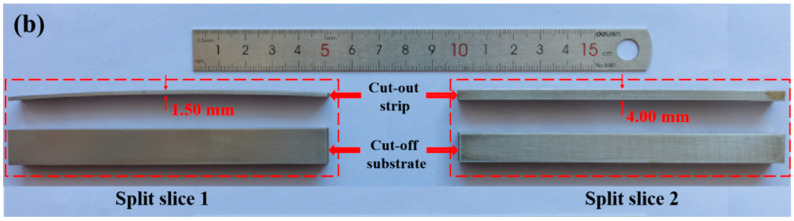
Split slices. (**a**) 45 steel; (**b**) 16Mn steel.

**Figure 8:**
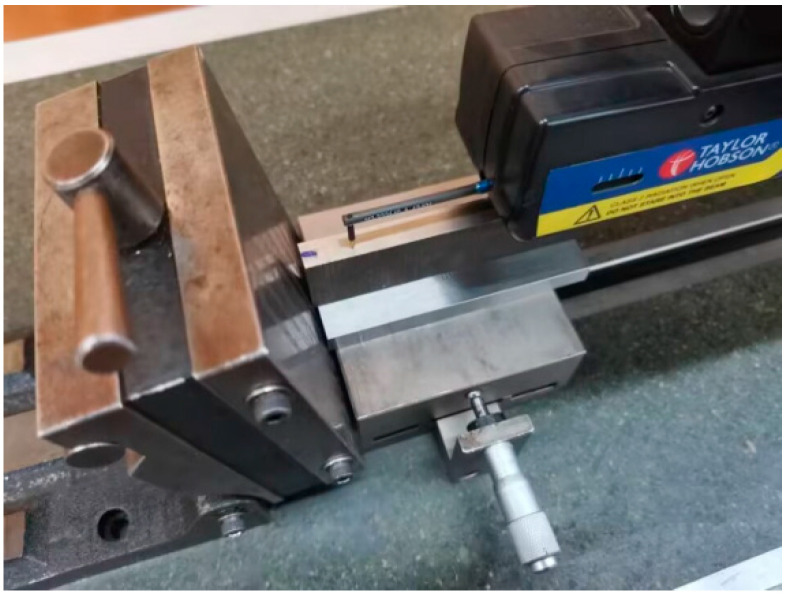
Measurement of curvature radius.

The 10 mm wide slice (*b* = 10.0 mm) is cut from a portion of the shot-peened specimen and a strip is cut from a portion of the slice. The shot-peened specimens, slice cutting and split slices are shown in Figure 5, Figure 6 and Figure 7. The measurement of curvature radius of strip and slice is shown in Figure 8. The elastic modulus (*E*) of both steels and the measured shape data of the narrow slices and strips are listed in Table 3. The corresponding values in Table 3 are substituted into Equation (9) to obtain a system of two equations with respect to *σ*_0_ and *t*_0_. By solving this system, *σ*_0_ and *t*_0_ are obtained. The calculated *σ*_0_ and *t*_0_ are 273 MPa (pressure stress) and 0.36 mm, respectively, for the shot-peened specimen of 45 steel, and 237 MPa (pressure stress) and 0.60 mm, respectively, for the 16Mn steel. The value of *t*_0_ is much smaller than *t* (*t*/*t*_0_ ratios of 4:1 and 8:1 for 45 steel, 3:1 and 7:1 for 16Mn steel), which fulfills the requirements.

This method is applicable only to flat specimens or those with a definite curvature radius. It determines the residual stress based solely on changes in the curvature radius of the surface-strengthened specimen after partial substrate removal. However, it is unsuitable for specimens with complex surface geometries, as the radius of surface curvature before and after strengthening cannot be accurately measured. Additionally, if the surface-strengthened specimen is thin, the cutting path may shift during partial substrate removal, leading to uneven strip thickness and resulting in measurement errors.

### 3.4. Comparison of the Results of Two Methods

For the convenience of comparison, the results of the two methods are listed in Table 4. In Table 4, the differences in the residual stress and the hardened layer’s thickness of the shot-peened specimen of 16Mn steel are both somewhat large. However, at present, no method is considered to be completely accurate in measuring residual stress. It is widely believed that the widely recognized X-/ECSM still has an uncertainty of ±32 MPa in measuring stress [26,27]. Considering this situation, the results of the two methods show acceptable agreement, which also show that the new method is feasible.

In short, the new method successfully determines the average residual stress and effective depth (surface-strengthened layer) for two material specimens through simple machining and measurement; it has been proven to be highly accurate.

The method of removing the constraint of the substrate has the advantages of convenient operation and an intuitive measurement process, but it also has obvious disadvantages, namely that the specimen is destroyed and the distribution of the internal stress cannot be measured. Furthermore, as noted above, while a suitable ratio range of *t*/*t*_0_ is necessary, the optimal bounds have not yet been established. In contrast, the X-/ECSM offers a significant advantage. It can determine the depth distribution of residual stress rather than just providing an average value over a layer thickness. However, as previously mentioned, it also has notable limitations.

## 4. Conclusions

A simple method of measuring the residual stress is designed, which belongs to the category of stripping methods and is suited for measuring the average residual stress and its effective depth in the surface-strengthened layer of a metal specimen.

(1) In the method, according to the principle of force equilibrium and the change in the radii of curvature of the surface-strengthened layer after stripping part of the substrate, the residual stress in the surface-strengthened layer and the thickness of the surface-strengthened layer could be obtained.
(2) The residual stress in the surface-strengthened layers and the thickness of the surface-strengthened layer of shot-peened specimens of 45 and 16Mn steels are measured using the new method and the widely recognized X-/ECSM. Although the results of the two methods are somewhat different, they still show acceptable agreement, which also shows that the new method is feasible.
(3) Compared to classical methods, the newly proposed method offers advantages such as simpler specimen preparation, better control over machining accuracy, and a more streamlined measurement procedure. However, it also has clear drawbacks: it is inherently destructive, cannot resolve the residual stress distribution, and is unsuitable for specimens with complex surface geometries.

## Figures and Tables

**Table 1:** Distribution of residual stress measured along the depth by X-/ECSM for the shot-peened specimen of 45 steel.

Depth, mm	Residual Stress, MPa		Average Residual Stress of Both Directions, MPa
	0° Direction	90° Direction	
0.00	−402.2	−382.0	−392.1
0.10	−333.3	−341.1	−337.2
0.21	−296.5	−281.0	−288.8
0.24	−231.3	−218.0	−224.7
0.26	−114.8	−119.8	−117.3
0.31	−36.8	−31.2	−34.0
0.40	+38.2	+30.8	+34.5
0.49	+58.3	+54.2	+56.3
0.54	+5.7	+9.4	+7.6

Note: Both directions were parallel to the exposed surface after corrosion. A positive value indicates tensile stress and a negative value indicates compressive stress.

**Table 2:** Distribution of residual stress measured along the depth by X-/ECSM for the shot-peened specimen of 16Mn steel.

Depth, mm	Residual Stress, MPa		Average Residual Stress of Both Directions, MPa
	0° Direction	90° Direction	
0.00	−311.1	−295.2	−303.2
0.06	−278.0	−338.5	−308.3
0.19	−268.4	−266.8	−267.6
0.24	−249.9	−195.6	−222.8
0.29	−209.2	−213.9	−211.6
0.33	−179.3	−162.7	−171.0
0.36	−175.9	−132.6	−154.3
0.40	−86.7	−90.2	−88.5
0.43	−56.4	−20.0	−38.2
0.48	−17.6	−60.2	−38.9
0.54	+46.8	+19.3	+33.1
0.60	+55.9	−16.7	+19.6

Note: Both directions are parallel to the exposed surface after corrosion. A positive value indicates tensile stress and a negative value indicates compressive stress.

**Table 3:** Measured shape data of the cut-out narrow slices and strips (*b* = 10.0 mm).

Specimens	*E*/10^6^ MPa	*T*_0_/mm	*R*_0_/mm		*t*/mm		*R*/mm	
			Slice 1	Slice 2	Strip 1	Strip 2	Strip 1	Strip 2
45	206,000	14.80	40,084.50	40,317.62	1.46	3.04	919.84	3205.00
16Mn	202,000	14.80	38,972.45	38,290.19	1.50	4.00	926.46	4595.87

Note: Strips 1 and 2 were cut from slices 1 and 2, respectively.

**Table 4:** Results of the two methods.

Specimens	X-/ECSM		RSCM		Difference, % (RSCM vs. X-/ECSM)	
	*σ*_0_, MPa	*t*_0_, mm	*σ*_0_, MPa	*t*_0_, mm	Residual Stress	Depth
45	256	0.35	273	0.36	6.6	2.9
16Mn	198	0.51	237	0.60	19.7	17.6

## Data Availability

The original contributions presented in this study are included in the article. Further inquiries can be directed to the corresponding author.
